# Mesenchymal Stromal/Stem Cells (MSCs) in Cancer Therapy: Advanced Therapeutic Strategies Towards Future Clinical Translation

**DOI:** 10.3390/molecules30244808

**Published:** 2025-12-17

**Authors:** Hanna Kucharczyk, Maciej Tarnowski, Marta Tkacz

**Affiliations:** Department of Physiology in Health Sciences, Faculty of Health Sciences, Pomeranian Medical University in Szczecin, Zolnierska 48, 70-210 Szczecin, Poland; hanna.kucharczyk77@gmail.com (H.K.); maciej.tarnowski@pum.edu.pl (M.T.)

**Keywords:** MSCs, cancer, cancer therapy, genetic modifications, drug carriers, MSCs bidirectional role

## Abstract

Mesenchymal stromal/stem cells (MSCs) appear in many studies, and their utilization is a developing area of study. Scientists are investigating the abilities of MSCs and the possibilities of using them in anticancer therapies, as well as combining such therapies with those currently used clinically. This article provides an overview of MSC-based therapeutic strategies, assessing their potential in the context of cancer treatment. These are engineering or biotechnological approaches that utilize the natural properties of MSCs in a targeted and therapeutically effective manner. The review focuses on innovative methods such as genetic modifications to express desired therapeutic molecules, highlighting their potential applications in clinical practice. Innovative strategies include modifications to express anticancer proteins, miRNA (microRNA), siRNA (small interfering RNA), lncRNA (long non-coding RNA), and circRNA (circular RNA) that induce specific effects, as well as the delivery of therapeutic genes and oncolytic viruses. However, further studies are required to address the existing impediments, which are also discussed in this review. A major challenge in the clinical application of MSCs is their bidirectional role, an issue that remains a central focus of current research and is examined in this article.

## 1. Introduction

According to World Health Organization (WHO) data, cancer is the most common cause of death worldwide. In 2022, cancer caused nearly 10 million deaths. In terms of new cancer cases, the most common was breast cancer, and in terms of deaths, lung cancer [1]. Cancer poses a critical challenge to public health all over the world [2]. For many decades, there were only a few available treatment options for patients, including surgery, radiation therapy, and chemotherapy, used as single treatments or in combination [3]. These primary treatment methods for cancer, with chemotherapy playing a crucial role in patient care, often face significant limitations. Despite its importance, chemotherapy’s effectiveness is often restricted by its lack of selectivity between tumor cells and normal cells. This can lead to inadequate drug concentrations in tumors, systemic toxicity, and drug-resistant tumor cells [4]. The rising occurrence of cancer and the insufficiency of current treatment methods have created a pressing need for the development of effective pharmaceuticals to treat various types of cancer [2,5].

Stem cells are unspecialized cells with the ability to differentiate and self-renew. They are found both in embryos and in adult tissues. Their differentiation potential varies depending on the type of stem cell and decreases with developmental stage. Mesenchymal stromal/stem cells (MSCs) are multipotent stem cells, which means that they have the ability to differentiate into cells of specific cell lineages. Specifically, they can specialize in mesodermal lineages (adipocytes, osteocytes, and chondrocytes). MSCs were first cultured from guinea pigs by Friedenstein as bone-forming cells. Later, Owen et al. expanded these studies to rats. In 1992, isolation of human bone marrow MSCs was reported, and infusion into patients was first carried out in 1993, as reported in 1995 [6].

The defining features of MSCs are immunomodulation, immunosuppression, genetic stability, poor immunogenicity, and their homing and tropism capacities. Due to these properties, they may be used in cell-based therapies for cancer treatment. MSCs can be isolated from tissues, such as adipose tissue, bone marrow, peripheral blood, menstrual blood, and neonatal tissues like the umbilical cord, placenta, amniotic fluid, and amniotic membrane [7,8,9,10]. When it comes to isolating MSCs, the methods can be classified based on cell types: bone marrow cells (BM-MSCs), peripheral blood cells (PB-MSCs), and adipose tissue cells (AT-MSCs). To prepare BM-MSCs for seeding, they should be centrifuged in a density gradient; then the mononuclear fraction is collected and seeded on a plastic dish for proliferation. AT-MSCs are obtained from biological material collected during liposuction, lipoplasty, or lipectomy. They are subjected to enzymatic digestion followed by centrifugation and washing. PB-MSCs can be obtained in two ways: (1) density gradient centrifugation followed by separation of the mononuclear cell fraction, or (2) separation of fibrin microbeads that were previously loaded with BM-MSCs [10]. According to the Mesenchymal Stromal Cell Committee of the International Society for Cell and Gene Therapy (MSC ISCT), the term mesenchymal stem cells does not have the same meaning as mesenchymal stromal cells. Mesenchymal stromal cells refer to a bulk population of cells with properties such as immunomodulation, homing, and secretory activity, whereas mesenchymal stem cells have to be able to self-renew and differentiate. Criteria to identify cells as multipotent mesenchymal stromal cells include the following: adherence to plastic surface during culture under standard conditions, expression of cell surface markers including CD29, CD44, CD49a-f, CD90, CD51, CD73, CD105, CD106, CD166, and Stro-1, lack of expression of CD45, CD34, CD14 or CD11b, CD79 or CD19, and HLA class II. A third criterion is the capacity for differentiation into adipocytes, chondrocytes, and osteoblasts in vitro [11,12].

In this review, we present the potential of MSCs in anticancer therapies and show that, due to their properties, they can be used not only as drug carriers but also in more advanced strategies. Delivery of oncolytic viruses (OVs) and expression of desired molecules, such as anticancer proteins, non-coding RNAs, suicide genes, and antiangiogenic genes, are described. We indicate possible limitations, the removal of which could enable future clinical applications. Equally important from a clinical perspective, MSCs exhibit both pro-tumor and tumor-suppressive effects within the tumor microenvironment. Therefore, we discuss their significant dual role, which influences anticancer therapies, as well as strategies to mitigate this risk using existing technologies.

It is also important to note that although valuable studies exist on this topic and provide a fundamental basis for understanding the role of MSCs in cancer therapies, the rapid development of research in this area means that many of their conclusions require critical updating with the latest data [5]. In contrast to previous studies, this article focuses primarily on innovative MSC-based anticancer therapeutic strategies, which merit detailed discussion because their use may contribute to the clinical development of anticancer therapies and represent the future of targeted cancer treatment. We discuss in detail novel molecular engineering strategies and genetic modifications of MSCs that have emerged from substantial research progress in this area. Furthermore, we provide a comprehensive listing of recent clinical trials, which are missing from other reviews. A comprehensive systematization of current knowledge on MSC-based therapies remains notably lacking. Our work not only addresses this need by integrating disparate findings from recent years but also significantly expands on existing knowledge by incorporating the latest findings.

## 2. Recruitment Mechanisms and MSC Activity in the Tumor Microenvironment

### 2.1. Tropism and Homing Abilities of MSCs

MSCs demonstrate tumor tropism, which is the ability to migrate selectively to target tissues, in this case, toward tumor sites. This process is mediated by various molecules, including cytokines, chemokines, and growth factors [13]. One of the best-known molecules involved in tropism is the chemokine SDF-1 (stromal cell-derived factor-1). SDF-1 binds to CXCR4 (chemokine receptor type 4) on MSCs, and in response, MSCs secrete more SDF-1. This autocrine effect induces MSCs’ migration toward the tumor site. Hypoxia also plays an important role. Tumors often do not receive an adequate amount of oxygen, which causes hypoxia. Under such conditions, pro-inflammatory mediators such as tumor necrosis factor-alfa (TNF-α), interleukin-6 (IL-6), or CCL-2 (also known as monocyte chemoattractant protein-1, MCP-1) are expressed. TNF-α, a pro-inflammatory cytokine, regulates the expression of cytokines, chemokines, and matrix metalloproteinases (MMPs), which are involved in MSCs’ migration. It has been shown that MSCs express CCR-2, which binds to CCL-2/MCP-1, thereby inducing MSCs’ migration [14,15,16]. When it comes to interleukins, tumor cells secrete IL-6, which binds to IL-6 receptor and Glycoprotein 130 (GP130) on MSCs. It activates STAT3 (Signal Transducer and Activator of Transcription 3) and induces secretion of CXCL-7 (chemokine (C-X-C motif) ligand 7). CXCL-7 interacts with tumor cells and promotes further synthesis of interleukins IL-6 and IL-8. In this way, the migratory phenotype of MSCs is enhanced. Growth factor PDGF (platelet-derived growth factor) forms dimers that bind to the PDGF receptor. This process activates the PI3K (phosphoinositide-3-kinase), Akt (protein kinase B), and ERK (extracellular signal-regulated kinase) pathways, resulting in MSCs’ migration toward the tumor. Another growth factor that impacts MSCs’ migration is PGF (placental growth factor), which is secreted by tumor cells in response to HIF-1α (hypoxia-inducible factor 1) expression. PGF binds to the VEGFR1 (vascular endothelial growth factor receptor 1) and induces the secretion of CXCL10 (chemokine (C-X-C motif) ligand 10) by MSCs. CCL5 is also secreted as a result of CSF-1 (colony-stimulating factor 1) binding to its receptor on MSCs. CXCL10 and CCL5 bind to CXCR3 (chemokine (C-X-C motif) receptor 3) and CCR5 receptors located on tumor cells. Moreover, MSCs stimulate sustained expression of HIF-1α. The entire process activates intracellular signaling and promotes tumor tropism [15,16,17].

**Figure 1:**
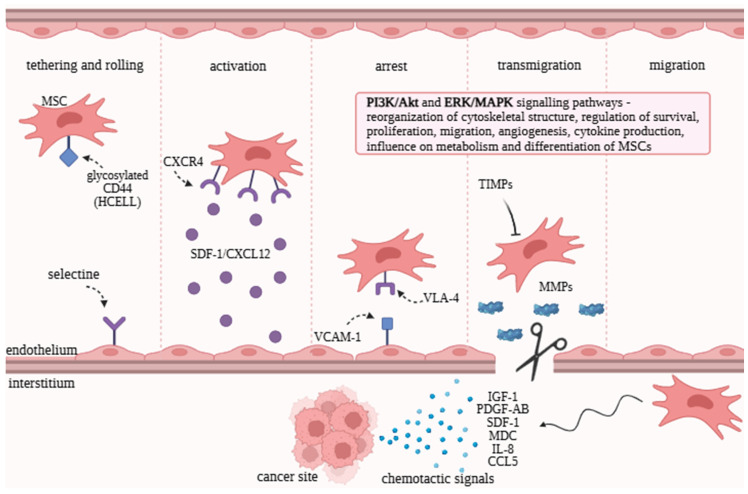
Mechanism of mesenchymal stromal/stem cells’ homing to tumor sites. The homing process of mesenchymal stromal/stem cells (MSCs) to cancerous tissue involves several distinct stages. CXCR4 (chemokine receptor type 4), SDF-1/CXCL12 (stromal cell-derived factor 1), VLA-4 (very late antigen-4), VCAM-1 (vascular cell adhesion molecule-1), MMPs (matrix metalloproteinases), TIMPs (tissue inhibitors of metalloproteinases), IGF-1 (insulin-like growth factor 1), PDGF-AB (platelet-derived growth factor-AB), MDC (macrophage-derived chemokine), SDF-1 (stromal cell-derived factor 1), IL-8 (interleukin-8), CCL5 (C–C motif chemokine ligand 5), PI3K (phosphoinositide 3-kinase), MAPK (mitogen-activated protein kinase), and ERK (extracellular signal-regulated kinase). Created with https://www.biorender.com/, accessed 20 October 2025.

Homing is a process involving a series of molecular interactions and mechanisms. Generally, it refers to the migration of cells to the tumor site. A more complex definition includes the active or passive arrest of MSCs in the vascular system, followed by transmigration through the endothelium. There are two types of homing: systemic and non-systemic (site-specific or local). Non-systemic homing does not involve vascular transport, whereas systemic homing does. Non-systemic homing requires either resident MSCs or transplantation of exogenous cells. Then, MSCs become activated and polarized, forming a leading edge (front pole). Directed migration is established, with the leading edge responsible for detecting chemokines released from injured tissue and guiding movement through the interstitial space. Migration ends when the target tissue is reached. Systemic homing is a multistep process that includes several stages and is preceded by the administration of MSCs into the bloodstream [18,19,20]. Homing is guided by a multitude of signals from various sources—selectins, chemokines, growth factors, and adhesion molecules—and different molecules are involved at each step (Figure 1).

In the first step, tethering and rolling, the most important molecules are selectins, which are expressed by endothelial cells. Selectins bind to the specialized glycoform of CD44 (HCELL). Functional HCELL expression on MSCs induces binding to endothelial E-selectin. It results in rolling along the vascular wall. Contact between MSCs and the endothelium slows down their flow within the bloodstream. This step is still under investigation. In the activation step, chemokines play an important role. MSCs express chemokine receptors that are coupled with G-proteins, and chemokines are secreted in response to inflammatory signals. The most crucial chemokine receptor is CXCR4, whose ligand is SDF-1/CXCL12. It has also been shown that MSCs express CCR1, CCR4, CCR7, CCR9, CCR10, CXCR5, CXCR6, and CXCR7. CXCR7 also binds SDF-1, which facilitates migration. The roles of the remaining chemokine receptors are still being investigated. The role of activation is important in the context of the next step because activation increases the affinity of integrins to their ligands. The third stage is arrest, in which integrins are involved. MSCs express integrins, and their ligands are located on the endothelium. In the previous step, as a result of chemokine action, the conformation of integrins changes to a high-affinity state. MSCs express VLA-4 (integrin a4β1), which binds to VCAM-1 (vascular cell adhesion molecule-1) on endothelial cells. The next step is transmigration/diapedesis—migration through endothelial cells and the basement membrane. In this process, MMPs play a role. They are lytic enzymes secreted by MSCs to degrade the endothelial basement membrane. MMP-2 (matrix metalloproteinase-2) and MMP-9 (matrix metalloproteinase-9) degrade collagen and gelatin, which are the main components. MMP activity is regulated by TIMPs (tissue inhibitors of metalloproteinases), whose expression is controlled by inflammatory cytokines. Thus, the entire process is induced by inflammation, which signals MSCs’ migration to the target tissue [19,20,21,22,23,24]. It has been shown that inhibition of MMP-2 decreased migration, whereas inhibition of TIMP-3 increases MSCs’ migration capacity [25]. The last stage is migration through the interstitium in damaged tissue. This process is controlled by chemotactic signals—appropriate molecules released in response to injury. These signals include chemokines, cytokines, and growth factors. MSCs migrate toward these signals and reach the target tissue [20].

### 2.2. Signaling Pathways and Their Role in Regulating MSC Functions

The key signaling pathway for the functioning of MSCs is the PI3K/Akt pathway. Factors regulating this pathway include various growth hormones, cytokines, and exogenous molecules. The key enzyme in this pathway, PI3K, converts phosphatidylinositol 4,5-bisphosphate into phosphatidylinositol 3,4,5-triphosphate, which binds both Akt and 3-phosphoinositide-dependent protein kinase 1 (PDK1), enabling PDK1 to phosphorylate Akt, its downstream target. Akt activation triggers a cascade of reactions that regulate various cellular functions [26,27].

The PI3K/Akt pathway regulates cell proliferation, apoptosis, differentiation, and migration. Also, PI3K/Akt activation is essential for MSCs to survive in hostile microenvironments such as hypoxia, oxidative stress, or nutrient deprivation [26,27].

Activation of the PI3K/Akt pathway has been shown to mediate the effects of several factors that enhance MSC proliferation. Its role in MSC proliferation has also been demonstrated by activating this pathway through mutations of the PI3K regulatory subunit in MSCs. The p85a subunit normally inhibits the p110 catalytic subunit; inactivation of p85α in MSCs results in increased levels of phosphorylated Akt. This is associated with increased cell numbers and enhanced colony formation. Akt also promotes cell proliferation by activating the mammalian target of rapamycin (mTOR) [27].

The PI3K/Akt pathway is well recognized for regulating cytoskeletal changes. The p70(S6K) protein can influence cytoskeletal dynamics through actin filament cross-linking proteins. Overexpression of p70(S6K) in ovarian cancer cells promotes directed cell migration. Inhibition of p70(S6K) activity results in reduced expression of these proteins and actin cytoskeletal reorganization [27].

In addition, the cytokine secretory capacity of MSCs is regulated by the PI3K/Akt pathway. This pathway also plays a key role in MSC differentiation, although its involvement is complex and highly context-dependent [27].

Activation of the PI3K/Akt pathway in MSCs has found applications in both cell therapy and tissue engineering. Stimulators of this pathway and overexpression of its key components have been shown to increase MSC efficacy. In tissue engineering studies, MSCs have been transplanted with various growth factors, such as VEGF and fibroblast growth factor (FGF), to enhance MSC functionality. The PI3K/Akt pathway has been shown to play a key role in the physiology and pathophysiology of numerous cell types, and its dysregulation contributes to the development of several serious disorders, including cancer [26,27,28,29].

A second key signaling pathway is the ERK/MAPK (extracellular signal-regulated kinase/mitogen-activated protein kinase) pathway. MAPK cascades are key signaling pathways that regulate a wide range of cellular processes: proliferation, differentiation, apoptosis, and stress responses. The MAPK pathway comprises three major kinases. ERK1 and ERK2, which are extracellular signal-regulated serine-threonine kinases that regulate cell signaling under both physiological and pathological conditions [30,31].

Activation of the MAPK/ERK pathway promotes migration and proliferation of MSCs [32]. This pathway is also a regulator of MSC differentiation and has been shown to stimulate both adipogenesis and osteogenesis through the phosphorylation of specific transcription factors. Importantly, it is the only signaling pathway that maintains activity across all three major MSC differentiation lineages: adipogenic, chondrogenic, and osteogenic [33]. Regarding migration, the MAPK/ERK pathway mediates MSC movement in response to factors such as SDF-1α or TNF-α [34].

However, ERK/MAPK signaling is also implicated in tumorigenesis. Elevated ERK expression has been detected in various human tumors, such as ovarian, colon, breast, and lung cancers. Increased activation of the ERK/MAPK signaling pathway can promote the transformation of normal cells into cancer cells, whereas inhibition of the ERK/MAPK signaling pathway can revert cancer cells to a non-transformed state in vitro and can inhibit tumor growth in vivo. These observations underline the therapeutic potential of targeting this pathway [30].

### 2.3. Bidirectional Regulatory Roles of MSCs in Tumors

MSCs play a dual and complex role in cancer pathophysiology through their capacity to either limit or promote tumor progression. They interact with the tumor microenvironment, modulate cancer cell behavior, influence their function, and promote distant metastasis through the secretion of mediators, regulation of cell–cell interactions, and modulation of the immune response. MSCs also exhibit therapeutic properties, including antitumor, anti-proliferative, anti-inflammatory, and antioxidant effects. Scientists also suggest that MSCs can undergo a functional transformation from an anti-tumor phenotype (MSC-1) to a tumor-promoting phenotype (MSC-2) [35].

MSCs are recruited to tumor sites for tissue repair but may be regulated by the pro-inflammatory TME, where they are exposed to numerous proinflammatory mediators and other stimuli that can alter their behavior and promote tumor development.

Within the tumor microenvironment, MSCs can differentiate into multiple cell types. They may generate fibroblasts that subsequently differentiate into cancer-associated fibroblasts (CAFs), which play a key role in tumor progression. MSCs can also differentiate into adipocytes, giving rise to cancer-associated adipocytes (CAAs), which actively contribute to the formation and function of the TME. Furthermore, MSCs may give rise to cancer stem cells (CSCs), characterized by the capacity to self-renew, differentiate, and the ability to reproduce cancer cell phenotypes [35].

With regard to immune regulation, MSCs exhibit contradictory functions, acting as both potential suppressors and activators of the immune response. When MSCs engraft into tissues with low levels of TNF-α and IFN-γ, they acquire a proinflammatory phenotype (MSC1) and secrete a large number of inflammatory factors (reactive oxygen species (ROS), IFN-α, IFN-β), enhancing the phagocytic properties of neutrophils and macrophages and the cytotoxicity of CTLs and NK cells. In contrast, exposure to high levels of inflammatory cytokines (TNF-α and IFN-γ) induces an immunosuppressive MSC2 phenotype characterized by increased production of anti-inflammatory factors (TGF-β, IL-10), which suppresses the effector function of inflammatory immune cells and attenuates ongoing inflammation. MSC2s also secrete PDL1 and PDL2, inhibiting T-cell proliferation and promoting the generation of immunosuppressive Treg cells [36].

The role of MSCs in regulating apoptosis is ambiguous and depends on the cytokine profile of the surrounding environment. Certain cytokine environments stimulate MSCs to exert anti-inflammatory, pro-survival effects, while others can have the opposite effect, increasing inflammation and promoting apoptosis. The protective effect of MSCs via inactivation of the PTEN suppressor results in activation of the PI3K/AKT survival pathway or inhibition of key pro-apoptotic mediators such as p53, JNK, and p38/MAPK. Conversely, MSCs have also been reported to promote apoptosis in cancer cells, including glioma, pancreatic cancer, hepatocellular carcinoma, and lymphoma [37].

Understanding this bidirectional nature of MSCs underscores the necessity of modifying them for therapeutic applications. The use of unmodified MSCs carries inherent limitations in both safety and efficacy. Therefore, engineering strategies, which are the focus of this work, are essential to mitigate these risks and advance the clinical translation of MSC-based anticancer therapies.

## 3. MSCs as Drug Carriers—Mechanisms of Drug Delivery by MSCs to Target Sites

A drug delivery system (DDS) refers to a set of physicochemical technologies that control the delivery and release of pharmacologically active substances into cells, tissues, and organs, including those with cancerous changes, in such a way that these substances exert optimal effects. DDS encompasses the routes of administration and drug formulations, aiming to maximize therapeutic efficacy while minimizing side effects [38]. MSCs have been developed as drug carriers. MSC-based drug delivery systems (MSC-DDS) are being investigated to improve disease treatment and overcome existing limitations [39].

MSCs occur not only in healthy tissues but are also found in the tumor microenvironment. Cancer cells can recruit healthy MSCs, which are then transformed into tumor-associated MSCs (TA-MSCs). Such modified cells become oncogenic and contribute to tumor progression. Targeting MSCs may therefore represent a therapeutic strategy, and their genetic modification can facilitate the elimination of tumor cells. In particular, MSC-derived exosomes, which act as intercellular communicators, appear promising in the prevention of tumorigenesis and in cancer treatment [17]. MSCs serve as carriers for drugs to the tumor site and damaged tissues. There are several approaches to load MSCs with drugs or drug-containing nanoparticles: surface loading, cellular internalization, or utilization of exosomes (the latter includes two strategies). It has been shown that surface loading of nanoparticles may be easier than cellular uptake. Modification of MSCs’ surface provides high-quality and rapid usability [40]. With regard to exosomes, their application is a promising treatment strategy. Exosomes are extracellular vesicles with a diameter of 40–100 nm that contain biomolecules such as proteins, nucleic acids, and lipids. Exosomes can be obtained from cultured MSCs, which enables large-scale production and their use for therapeutic purposes. MSC-derived exosomes contain molecules similar to those of the parent cells, allowing them to mimic key properties of MSCs, including immunomodulatory functions and the ability to influence target cells in a comparable manner. Because they inherit a wide range of bioactive components from MSCs, they exhibit functions closely resembling those of the originating cells. These extracellular vesicles (EVs) are characterized by biocompatibility, low immunogenicity, minimal oncogenic potential, a favorable safety profile, and the capacity to cross biological barriers, all of which enhance their therapeutic potential [41,42,43]. One strategy involves isolating exosomes from MSCs, loading them with a therapeutic agent, and subsequently administering them to the patient or reintroducing them into cells. Another approach is to deliver bioactive substances into exosomes during their biogenesis, enabling the vesicles to be secreted with the drug naturally encapsulated and targeted to the desired site. It has been demonstrated that nanodrugs loaded into exosomes can effectively treat osteosarcoma and exert therapeutic effects. Moreover, MSC-derived exosomes may modulate liver fibrosis, inhibit inflammation, and contribute to immunoregulation [17,44].

Exosome formation occurs in several stages. Initially, early endosomes originate from invaginations of the cell membrane, generating small vesicular structures. These subsequently mature into late endosomes, during which specific proteins and lipids are incorporated into their membranes. Further maturation leads to the development of multivesicular bodies (MVBs), within which intraluminal vesicles (ILVs) form. ILVs eventually become exosomes, which are released upon fusion of the MVBs with the plasma membrane.

Exosomes are capable of delivering receptor ligands, enzymes, cytokines, and genetic material from the parent cell. After entering target cells, they transmit signals that may act as inhibitors or activators, induce phenotypic changes, and promote genetic reprogramming. MSC-derived exosomes exert effects through immunomodulation, anti-apoptotic activity, facilitation of cellular communication, tissue repair and regeneration, and modulation of extracellular matrix degradation. They can regulate immune responses, deliver anti-apoptotic factors or suppress pro-apoptotic genes, stimulate cell proliferation and differentiation, and transport bioactive molecules to support intercellular communication [42,43].

As described in the previous chapter, MSCs possess tumor tropism, which enables drug delivery to the site of pathology. MSCs used as drug carriers migrate to target tissues in response to factors secreted by the diseased area, ensuring selective transport [20]. Ongoing research aims to improve the targeting efficiency of MSC-based drug delivery, including surface modification of MSCs’ exosomes or the application of peptide conjugates. Studies have focused on breast, lung, and liver cancers [44].

The release of the exosomes from MSCs occurs through the fusion of MVBs with the plasma membrane. The subsequent release of substances (including drugs) into the extracellular space at the target site can occur through several mechanisms. One involves internalization of exosomes by the target cell via phagocytosis, endocytosis, or pinocytosis. Another mechanism involves fusion of the exosome membrane with the target cell membrane or ligand–receptor interactions on the cell surface [45,46].

Another, less well-understood, form of intercellular communication involves tunneling nanotubes (TNTs)—cytoplasmic extensions supported by F-actin filaments that form open-ended channels enabling cell-to-cell communication over long distances. Cytoplasmic material can also be exchanged through these structures. A specific set of proteins is responsible for the process of nanotube formation. The M-Sec protein and the RalA molecule play a key role here, acting by initiating tube construction. Transport within nanotubes is an active process and relies on motor proteins that function as engines for moving cargo. Myosin 10 (Myo10) is particularly important, and studies have shown that it is essential for vesicle transport within the tube. Increasing the cellular level of Myo10 results in faster and more efficient cargo transfer [47,48].

TnTs are classified into type I TnTs, which are short, dynamic structures containing actin filaments and involved in exploring the surrounding microenvironment, and type II TnTs, which are longer, more stable processes containing actin and tubulin filaments and involved in organelle transport. Studies indicate that TNTs may potentially be useful for drug delivery in cancer therapy [49].

Contemporary research on TNT applications focuses on their dual role. TNTs have been shown to serve as a pathway for mitochondrial transfer between cancer cells. Mitochondrial TNT-mediated transfer occurs in many cellular microenvironments, including tumors, and participates in a wide range of physiological and pathological processes, such as immune responses, cell proliferation and apoptosis, substance transport, and angiogenesis. Although mitochondrial transfer within the tumor microenvironment typically promotes tumor progression, introducing healthy mitochondria into cells from the outside can have the opposite effect. However, some studies have shown that artificial transfer of mitochondria to cancer cells can promote tumor growth, seemingly contradicting other findings. This inconsistency highlights the need to systematize research in order to use this strategy clinically [50].

Another strategy aims to overcome the possible tumor-promoting effect of TNTs. It is based on using TNTs as drug distribution channels, facilitating the intercellular transfer of nanoparticles and cytostatic drugs. A key advantage of this approach is the ability to improve the biodistribution of therapeutics within solid tumors. Owing to the TNT network, a drug introduced into one cell can be transported to deeper, poorly vascularized tumor niches. This approach effectively transforms a mechanism of tumor invasiveness into a tool for combating it [51].

Despite their promising therapeutic potential, clinical translation of TNT-based strategies faces technical limitations. Although visualizing TNTs in cells by labeling the cytoskeleton appears straightforward, TNTs display diverse and complex structures, ranging from those containing only single actin filaments to those containing both actin filaments and microtubules. They also vary in length and thickness, with no strict criteria distinguishing them from similar structures such as filopodia and invadopodia. Furthermore, TNTs are highly sensitive to mechanical stress, chemical fixatives, and light, making it difficult to determine their true structure in their native state. The sensitivity and fragility of TNTs require extremely high-resolution imaging [52].

Numerous preclinical studies on MSC-based cancer therapies are currently underway [40,53]. MSCs may act as carriers of cytostatics—anticancer drugs used in chemotherapy. In both in vitro and in vivo assays, MSCs loaded with drugs have demonstrated anticancer effects [53,54]. Table 1 shows the studies presented below.

Efficient drug loading is a critical parameter determining the therapeutic performance of MSC-based delivery systems. These methods differ in efficiency, scalability, and impact on cell viability, which poses a major limitation in achieving predictable and reproducible therapeutic outcomes [60]. MSC-based drug loading occurs through multiple approaches, including endocytic uptake, surface anchoring, and direct loading via physical or chemical methods such as electroporation or sonication [61,62]. Anti-cancer agents or drug-loaded nanoparticles (NPs) are introduced into the MSC cytoplasm through passive diffusion or endocytic uptake. A dual drug-loading strategy integrating endocytic uptake and membrane-associated loading has also been employed to maximize the intracellular accumulation of DOX conjugates in MSCs [61,62]. In vivo, as reported by Yao et al., loaded MSCs were found to preferentially localize and persist in the mice’s lungs, which corresponded to sites of metastatic lesions [62]. Another strategy involves the membrane-engineering approach, which can be divided into three main categories: lipid-mediated insertion or fusion, biomarker-dependent anchoring, and chemical surface modification [62]. Kono et al. successfully achieved efficient and harmless loading of liposomes onto the surface of MSCs using large-sized Mag-AL complexes. They demonstrated that the resulting Lip-MSCs retained in vitro adhesion and tumor-homing capabilities comparable to those of unloaded MSCs [63].

Release kinetics is another critical factor influencing therapeutic performance. As demonstrated for MSC-derived EVs, it is essential to evaluate and compare the efficacy of different administration routes, since they significantly impact treatment outcomes [64]. The use of drug-encapsulated nanoparticles that provide sustained release can enhance the retention of the drug within the cell [61]. It was reported that loading DOX onto polyamidoamine dendrimers (DOX-PAMAM) was shown to delay its release from MSCs by 12–48 h compared with free DOX [61]. The in vivo stability of MSC-based drug delivery systems, ensuring sufficient persistence and maintenance of cargo integrity, is fundamentally important for maximizing therapeutic efficacy. Following intravenous administration, culturing MSCs as 3D spheroids for 48 h reduced their cell diameter by 34.6% and significantly enhanced their ability to traverse the lungs and migrate to other organs, such as the liver [65]. In turn, a thermosensitive hydrogel can serve as a sustained-release carrier to improve hUCMSC-EV retention and bioavailability, enhancing their therapeutic efficacy for IUA by prolonging persistence in the uterine environment [66]. Despite progress, variability in loading and release efficiency, inconsistent in vivo stability, and MSC/EV heterogeneity remain key translational barriers. Modern strategies, such as the use of carriers (e.g., hydrogels) or surface engineering, appear promising but require further systematic in vivo studies, potency testing, and procedural standardization.

Despite the fact that MSCs isolated from various tissues meet the minimum identification criteria defined by ISCT, the scientific literature increasingly indicates their functional heterogeneity depending on the source of origin [67]. It has been shown that MSCs derived from different tissues differ in their differentiation capacity, even when cultured under the same culture conditions [68].

Selecting the optimal source of MSCs is a crucial step in developing effective anticancer therapies. To provide a benchmark for clinical selection, Table 2 summarizes the key characteristics of MSCs derived from bone marrow (BM-MSCs), adipose tissue (AT-MSCs), and peripheral blood (PB-MSCs). The comparison includes clinical availability, proliferative potential, and specific biological properties such as homing efficiency, drug internalization capacity, and immunogenicity profile.

## 4. Innovative Therapeutic Strategies Using MSCs

The potential of engineered MSCs as an innovative and effective cancer therapy strategy stems from their ability to provide localized delivery, thereby minimizing systemic toxicities and enhancing therapeutic outcomes [76]. MSCs exhibit an inherent tropism toward sites of tissue damage and neoplastic lesions, positioning them as a compelling cellular platform for the targeted delivery of therapeutic agents to tumors [77]. Advanced cancer treatment strategies encompass immunotherapy, targeted therapy, gene therapy, and photodynamic therapy [77].

Genetic engineering aims to improve the therapeutic efficiency of MSCs, as their limited survival, retention, and engraftment hinder clinical application. To enhance these innate properties, MSCs are genetically modified using either viral or non-viral methods [78]. Viral transduction remains the most efficient technique for integrating exogenous genes into MSCs, achieving up to 90% transfection efficiency. An important advantage of this method is that it does not affect the quality and or differentiation potential of progenitor cells. The most commonly used viral vectors are adenoviruses, lentiviruses, and adeno-associated viruses (AVVs). However, viral transfection carries certain risks such as activation of oncogenes, induction of immune reactions, and loss of transgene stability [78,79]. Accordingly, Benabdallah et al. argue that targeted integration can minimize these adverse effects. In their study, zinc-finger nucleases (ZFNs) were delivered into MSCs using adenoviral vectors, and the erythropoietin (Epo) gene was introduced via lentivirus into the CCR5 locus. Mice receiving the modified MSCs exhibited higher Epo and hematocrit levels compared to controls [80].

Non-viral transduction involves introducing genes into MSCs using physical or chemical methods. Physical methods include electroporation and ultrasound sonoporation. Electroporation temporarily opens cell pores using electric pulses to facilitate the transfer of genetic material into the cytoplasm, though it may cause significant cell death. Sonoporation, based on mechanical vibration, increases cell membrane permeability and enhances nucleic acid uptake [78,81]. Using ultrasound and microbubbles has also been shown to enable efficient delivery of siRNA to MSCs [82]. Chemical methods utilize lipid agents, polymeric carriers, or inorganic nanoparticles. The highest reported efficiency (~80%) was achieved by Muroski et al., who used gold nanoparticles modified with Ku70 peptides [83].

### 4.1. Delivery of Oncolytic Viruses by MSCs

Oncolytic virotherapy is based on the selective degradation of cancer cells using oncolytic viruses (OVs). OVs enhance therapeutic efficacy and reduce the limitations associated with systemic administration. Researchers have developed an advanced strategy employing MSCs as carriers to transport oncolytic viruses to tumor sites. MSCs possess several crucial properties that make them suitable for this approach: homing and tropism abilities, immunomodulatory capacity, and protection of viruses from immune neutralization and degradation [84,85]. The combination of MSC and OV properties may therefore represent a powerful and innovative approach to cancer therapy [86]. Moreover, certain viruses can replicate within MSCs, further increasing the therapeutic potential. Examples of DNA oncolytic viruses include adenoviruses, herpes simplex virus (HSV), parvoviruses, poxviruses such as vaccinia virus (VACV) and myxoma virus (MYXV). Examples of RNA oncolytic viruses include coxsackievirus, Newcastle disease virus (NDV), maraba virus, measles virus (MV), poliovirus, reovirus, and retroviruses [87].

Oncolytic viruses exert their effects through two main mechanisms: cancer cells’ death and activation of antitumor immunity; they can also replicate within tumor cells. They not only induce lysis of cancer cells but also activate the immune response. The combination of these actions results in a strong anti-cancer effect. The virus can be introduced into MSCs ex vivo, and after administration, the infected cells migrate and deliver the virus to the target site. However, the precise mechanism of OV release by MSCs within tumors has not yet been fully described in the available literature. Cells infected by oncolytic viruses undergo cytotoxic destruction, and newly replicated viruses are released to infect additional tumor cells, promoting further oncolysis [88]. Oncolytic viruses can also induce apoptosis or immunogenic cell death (ICD) in cancer cells [89].

Oncolytic viruses recognize surface markers like CD20 or CD46 and endothelial growth factor receptors, which are expressed by cancer cells. Other relevant receptors include junctional adhesion molecules-A (JAM-A) and intracellular adhesion molecule-1 (ICAM-1). It has been discovered that the measles virus selectively targets tumor cells expressing CD150 and CD46, while the herpes simplex virus (HSV) interacts with HVEM, nectin-1, and nectin-2 receptors, facilitating viral entry [90,91,92]. After entering the cancer cell, the oncolytic virus delivers its genome to the nucleus, where viral replication occurs. New virions and transgene-encoded proteins are subsequently produced [93]. OVs work selectively because cancer cells exhibit impaired antiviral defense mechanisms. In healthy cells, the interferon pathway blocks viral replication in response to the secretion of type I interferons, activating antiviral responses and inducing apoptosis. In contrast, cancer cells often display defective IFN-I signaling, rendering them more susceptible to viral infection. Likewise, the tumor suppressor gene p53, which induces apoptosis in healthy cells, is frequently inactivated in cancer cells, allowing efficient viral replication. Adenoviruses normally express E1B-19K, which inhibits apoptosis, and E1B-55K, which degrades p53, thereby further suppressing apoptosis. To enhance safety and avoid damaging healthy cells, modified adenoviral variants with deletions in the E1B gene have been developed [86,92,94]. Dysregulation of signaling pathways such as RAS or PI3K/Akt/mTOR also contributes to increased susceptibility of tumor cells to oncolytic viral action. Ultimately, cell lysis occurs, releasing new virions, tumor-associated antigens (TAAs), and molecules that modulate the immune response [91]. It has been found that the disintegration of cancer cells caused by OVs also leads to the release of molecules that stimulate the innate immune response. Tumor cells release PAMPs (pathogen-associated molecular patterns) and DAMPs (damage-associated molecular patterns), which act as danger signals to the immune system. PAMPs include viral nucleic acids and proteins, while DAMPs include heat shock proteins (HSPs), high-mobility group box 1 (HMGB1), and calreticulin (CRT). Immune cells such as natural killer (NK) cells and macrophages recognize these signals and, in response, secrete inflammatory cytokines (e.g., IFN-_Y_, IL-12, IFN-a, TNF-a, IL-6) [89,95]. Dendritic cells (DCs) are also recruited, mature, and present tumor-associated antigens (TAAs) to cytotoxic T lymphocytes (CTLs) [95,96]. This process leads to the activation of both innate and adaptive tumor-specific immunity [97,98].

Preclinical and clinical studies are currently underway using MSCs as OVs carriers [88]. For instance, HSV has been tested in malignant glioblastoma multiforme mouse models, demonstrating tumor destruction and prolonged survival [99]. Similarly, studies involving Newcastle disease virus in HPV (human papillomavirus) associated tumors (mouse model) have shown reduced tumor growth, induction of immune responses, and increased expression of apoptotic proteins [100]. MSCs as carriers of oncolytic viruses represent a promising cancer treatment strategy. These cells exhibit tumor-directed migration and rapid replication in culture, providing a practical advantage for therapeutic application [101]. However, several challenges remain, including risk of a proinflammatory environment [102], potential resistance of cancer cells, and uncontrolled viral replication within MSCs [103].

To overcome these issues, research teams have developed modified oncolytic viruses to improve therapeutic efficacy. For example, the use of the conditionally replicative adenovirus (CRAd) was investigated in glioblastoma. Scientists employed a Tet-On system regulating viral replication via doxycycline (DOX) and incorporated therapeutic genes IL-24 and endostatin (ES). The results showed optimized MSC viability, increased viral loading capacity, enhanced tumor gene therapy efficacy, and stronger antitumor activity against glioma [104]. Another study examined the adenovirus ICOVIR-5 in metastatic neuroblastoma. The formulation known as CELYVIR- autologous MSCs infected with ICOVIR-5 that selectively replicate in cancer cells demonstrated good treatment tolerance and even complete remission in one patient [105].

### 4.2. Molecular Engineering Strategies to Improve the Anticancer Potential of MSCs

The CRISPR/Cas (Clustered Regularly Interspaced Short Palindromic Repeats–associated protein) system enables precise genome editing at specific DNA loci [106,107]. In addition to the Cas nuclease, this technology requires a guide RNA (gRNA) that directs the Cas enzyme to the target site [108]. CRISPR/Cas allows for oncogene inactivation, enhancement of immune responses, correction of gene mutations, and delivery of cancer-killing molecules [109]. This system has been applied to improve the therapeutic efficacy of MSCs. After CRISPR/Cas modification, MSCs can be administered intravenously or intratissue, depending on the experimental design [110]. Ongoing research continues to optimize the CRISPR/Cas method to improve editing efficiency, cell survival, and immunomodulatory activity [111].

However, the field is currently shifting towards more precise gene editing tools, such as base editing and epigenetic editing, whose application may be significant for MSCs engineering.

Base editors developed on the CRISPR/Cas platform have shown great potential because, unlike conventional CRISPR/Cas systems, they do not generate double-strand breaks (DSBs) while enabling precise single-base substitutions. It has been shown that base editing does not perturb the transcriptional profile of edited cells, even within DNA repair pathways, in contrast to double-strand break-based strategies. This feature may offer important advantages for the clinical application of base editors. Despite the considerable potential of base-editing technology, this therapeutic approach still requires substantial development before it can transition from laboratory studies to clinical use [112,113].

Epigenetic regulation refers to processes that cause heritable changes in gene expression without altering the underlying DNA sequence. These processes include DNA methylation, histone modifications, and the regulation of non-coding RNA. Epigenetic mechanisms play a key regulatory role in MSCs’ biological behaviors, such as homeostasis, cell senescence, cell proliferation, and cell death [114]. Understanding these mechanisms has led to the development of epigenetic editing tools that enable direct control over gene activity. Epigenetic editing relies on CRISPR–dCas9 (catalytically deficient Cas9) and involves the targeted recruitment of epigenetic enzymes to modify the epigenetic code and reprogram transcription. This approach has substantially accelerated the development of epigenetic manipulation and has already demonstrated preclinical therapeutic benefits using a range of epigenetic enzymes [115].

Gene editing is used both to study and enhance MSC functionality, yielding superior therapeutic outcomes compared with unmodified MSCs. Such molecular engineering strategies enable precise modulation of their biological properties. Genetic modifications can improve both clinical efficacy and therapeutic safety [78]. However, despite the considerable promise of these tools, many challenges remain before their full potential can be realized [116].

### 4.3. Expression of Anticancer Proteins

**Figure 2:**
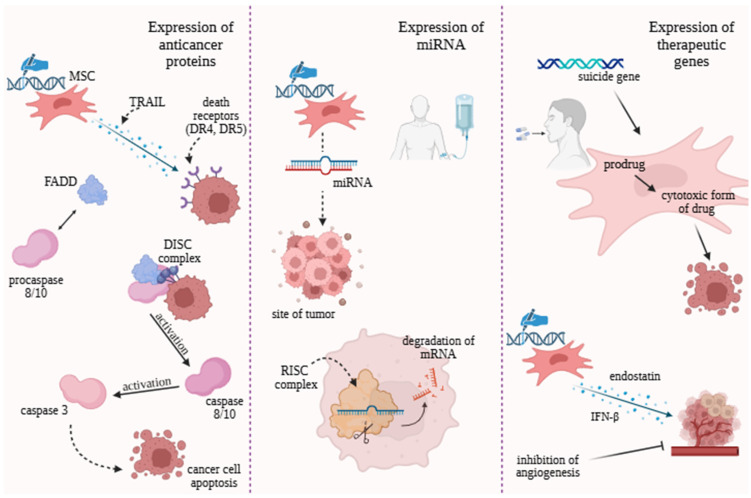
Expression of miRNA, anticancer proteins, and therapeutic genes as innovative strategies in MSC-based cancer therapies. MSC (Mesenchymal stem/stromal cells), TRAIL (Tumor necrosis factor-related apoptosis-inducing ligand), DR4 (Death receptor 4), DR5 (Death receptor 5), FADD (Fas-associated death domain protein), DISC (Death-inducing signaling complex), RISC (RNA-induced silencing complex), IFN-β (Interferon beta). Created with https://www.biorender.com/, accessed 20 October 2025.

MSCs can be genetically modified to express therapeutic proteins, enabling targeted anticancer therapy. One of the best-studied examples is the tumor necrosis factor (TNF)-related apoptosis-inducing ligand (TRAIL)—a cytokine that induces apoptosis in cancer cells by binding to the death receptors DR4 and DR5 on the target cell surface [81]. Upon ligand binding, receptor trimerization occurs, activating a downstream signaling cascade. The FADD (Fas-associated death domain) adaptor and procaspases 8/10 are recruited to form the DISC (death-inducing signaling complex), which activates caspase-8/10, subsequently triggering caspase-3–mediated apoptosis (Figure 2) [81]. Studies have shown that TRAIL-overexpressing MSCs inhibit tumor growth in H460 non-small cell lung cancer xenograft models [117]. Further experiments have combined TRAIL-modified MSCs with chemotherapeutic agents (e.g., gemcitabine in pancreatic ductal carcinoma or cisplatin in cervical cancer), demonstrating synergistic anticancer effects, enhanced therapeutic efficacy, and confirmed pro-apoptotic activity of TRAIL [118,119]. Although TRAIL-expressing MSCs represent a promising approach, certain challenges remain—notably, cancer cell resistance to TRAIL-induced apoptosis [120], often due to low death receptor expression or upregulation of anti-apoptotic proteins [121]. A deeper understanding of cancer stem cell (CSC) mechanisms and resistance pathways is crucial for advancing MSC–TRAIL-based therapies (Figure 2).

Genetically modified MSCs can be engineered to secrete immunostimulatory cytokines within the tumor microenvironment (TME), thereby activating immune responses. Cytokines are essential mediators of communication between immune and stromal cells [122]. For example, MSCs engineered to express IL-12 have demonstrated inhibition of primary and metastatic tumor growth in murine models, accompanied by reduced vascular density and increased infiltration of anticancer macrophages and cytotoxic T lymphocytes [123]. Earlier studies investigated MSCs expressing interferons (IFNs)—α, β, and γ—as carriers in various cancer models: MSC–IFNβ combined with temozolomide in glioma therapy [124]; MSC–IFNα in melanoma lung metastasis [125]; MSC–IFNγ in chronic myelogenous leukemia [126]. All these studies demonstrated anticancer potential, although the number of contemporary studies remains limited, highlighting opportunities for further investigation.

### 4.4. Engineering Strategies Based on Non-Coding RNAs Modulation

MicroRNAs (miRNAs) are short, non-coding RNA molecules that regulate post-transcriptional gene expression [127]. They associate with the RNA-induced silencing complex (RISC), which recognizes complementary sequences within the 3′ untranslated region (3′UTR) of target oncogene mRNAs, leading to mRNA degradation or translational repression (Figure 2) [128]. Studies suggest that MSCs-modified to express therapeutic miRNAs can exert direct antitumor effects at the tumor site, demonstrating promising therapeutic potential [129]. Table 3 (below) summarizes studies describing the effects of various miRNAs on cancer cells.

MSCs can be genetically modified to express small interfering RNA (siRNA). These small molecules, which are delivered exogenously to the cell, work through RNAi (RNA-interfering) pathways. This process involves the specific silencing of targeted gene expression. There is potential to utilize this strategy for targeted cancer therapy [140,141]. The mechanism of action of siRNA is based on its binding to the RISC (RNA-induced) silencing complex, where one of the siRNA strands guides RISC to mRNA. This results in degradation of the target mRNA and suppression of specific gene expression. It has been demonstrated that siRNA can be delivered effectively to selectively silence genes [142]. Scientists face many difficulties when it comes to delivering siRNA to cancer cells using MSCs. For this reason, new methods are being developed to overcome these issues. Biomimetic nanovesicles with genetically modified cell membranes have been designed. Results show improved delivery of siRNA and inhibition of metastasis and tumor growth by silencing the EGFR (epidermal growth factor receptor) gene [143]. Recruitment for a phase clinical trial is being conducted (NCT03608631) on exosomes derived from MSCs loaded with siRNA against KrasG12D in metastatic pancreatic ductal adenocarcinoma (PDAC) [144]. Downregulation of the Survivin gene, an inhibitor of apoptosis in tumor cells, using siRNA has also been investigated, showing a reduction in tumor growth [145]. Strategies using MSCs modified to express microRNA and siRNA are promising in cancer therapy. Nevertheless, more studies have focused on miRNA than siRNA. This may be due to the endogenous nature of miRNA, which allows natural regulation, and its ability to target multiple genes simultaneously [146].

Long non-coding RNAs (lncRNAs) are key regulators of stem cell differentiation, acting as important modulators of gene expression. By coordinating stem cell fate decisions and lineage determination through epigenetic, transcriptional, and post-transcriptional mechanisms, lncRNAs have emerged as critical determinants of cellular behavior. They function primarily by interacting with specific proteins, altering their activity and thereby modulating downstream gene expression. Through these interactions, lncRNAs significantly influence MSCs’ biology, particularly in directing lineage commitment and differentiation. Their potential interactions with microRNAs have also been demonstrated. LncRNAs can act as competing endogenous RNAs, interfering with miRNAs and preventing them from repressing their target mRNA. As a result, translation of genes normally silenced by the corresponding miRNA is restored. Such a process is called miRNA sponging [147]. Circular RNAs (circRNAs) also act as miRNA sponges and regulators of gene expression. CircRNAs are a class of RNAs characterized by a closed-loop structure, which confers enhanced stability.

However, these types of RNAs have been implicated in the development and regulation of CSCs. Aberrant expression of circRNAs and lncRNAs in CSCs may contribute to oncogenic properties and drug resistance, as well as enhanced self-renewal and maintenance, thereby promoting cancer progression. Investigating circRNAs and lncRNAs as potential therapeutic targets offers a promising avenue for developing more selective and effective anticancer strategies.

In the context of MSC engineering, the described molecular mechanisms open new possibilities for controlling cell behavior. Precise modulation of lncRNA and circRNA allows not only the direction of MSC differentiation but also the targeting and elimination of cancer cells, which is crucial for therapeutic efficacy. Moreover, circRNAs show promising diagnostic, prognostic, and predictive value as biomarkers [148,149,150].

### 4.5. Therapeutic Genes

Gene-directed enzyme prodrug therapy (GDEPT) is a strategy in which cancer cells are transduced with a gene encoding a non-toxic enzyme, known as a suicide gene. This enzyme converts a prodrug into its active, cytotoxic form, resulting in cancer cell death (Figure 2) [151]. The efficacy of this approach was evaluated in glioblastoma multiforme in rats, where human MSCs were modified to express the yCD::UPRT gene. Exosomes derived from these MSCs delivered mRNA encoding an enzyme that converts the prodrug 5-fluorocytosine into its active form, 5-fluorouracil. The results demonstrated inhibition of tumor growth, and most of the treated rats achieved complete remission [152]. Another study confirmed similar effects in glioma, showing the killing of both glioma cell lines and primary human glioblastoma cells. MSCs transduced with the suicide gene herpes simplex virus thymidine kinase (HSVTK) secreted exosomes containing mRNA, which enabled the conversion of the prodrug ganciclovir (GCV) to its active form, GCV-triphosphate, inducing cell death [153]. An additional strategy involves genetic modifications of MSCs to produce anti-angiogenic factors. MSCs engineered to deliver human endostatin to the site of tumor were tested in a murine model of peritoneal ovarian cancer. The results revealed a reduction in tumor volume without systemic toxicity, accompanied by decreased vascular density, reduced tumor cell proliferation, and increased apoptosis [154]. Although this area remains under investigation, later studies have confirmed these findings [104]. The anticancer effects of MSC-delivered endostatin have also been observed in other diseases, supporting further exploration of this therapeutic approach [155]. Another anticancer factor is interferon beta, a molecule capable of inducing apoptosis, inhibiting angiogenesis, and recruiting T cells. These effects have been demonstrated in a murine model of glioma [156].

### 4.6. Advantages and Disadvantages of Various Innovative Therapeutic Strategies

MSCs have emerged as a promising vehicle for advanced anticancer approaches owing to their tumor-homing capacity, immunomodulatory functions, and suitability for genetic and pharmacological engineering. Several innovative strategies, including genetically modified MSCs, drug-loaded MSCs, MSC-mediated oncolytic virotherapy, and MSC-derived extracellular vesicles (EVs), constitute an intensive advancing area of research in anticancer therapy. However, despite their promising potential, each approach carries distinct advantages and limitations that influence translational feasibility, safety, and therapeutic consistency.

MSCs as carriers of oncolytic viruses represent one of the most promising strategies of cancer treatment. These cells exhibit tumor-directed migration and rapid replication in culture, providing a practical advantage for therapeutic application [101]. Nevertheless, several challenges remain, including risk of a proinflammatory environment [102], potential resistance of cancer cells, and uncontrolled viral replication within MSCs [103]. Viral transduction is still the most effective method for delivering exogenous genes into MSCs, achieving transduction efficiencies approaching 90%. A key advantage of this method is that it preserves both the functional and the differentiation capacity of progenitor cells. However, viral gene delivery is associated with risks, including potential oncogene activation, induction of immune responses, and loss of transgene stability [78,79]. Viral vectors can trigger immune reactions or insertional mutations that can lead to oncogenesis, while non-viral approaches frequently result in increased cell death or only transient transgene expression [157,158].

Genetically modified MSCs can express therapeutic proteins, thereby facilitating targeted anticancer therapy [81,117,124,153]. However, due to the high heterogeneity of MSC populations (different subpopulations, sources, and culture conditions), standardizing procedures and predicting therapeutic outcomes remain challenging [159].

MSC-based drug delivery systems (MSC-DDS) are being explored to improve disease treatment and address existing limitations [39]. An undeniable advantage of MSC-based therapy is its tumor-homing capacity and reduced systemic toxicity due to targeted delivery. Moreover, they can transport drugs that are difficult to solubilize or are stable only within specific carriers [60]. The challenge, however, lies in the variable efficiency of drug loading and release, the potential pro-tumor effects of MSCs within the tumor microenvironment, and the difficulties in standardizing both cell and carrier production [76].

An attractive alternative to based therapy is the use of EVs, which have emerged as a promising cell-free therapeutic approach for immunomodulation [160]. EVs show biocompatibility, low immunogenicity, minimal oncogenic risk, a favorable safety profile, and the ability to cross biological barriers, collectively enhancing their therapeutic potential [41,42,43,161]. However, MSC-derived EVs face significant barriers to clinical translation. There is high variability in EV preparations (depending on the MSC source, isolation protocol, and cargo), which complicates standardization and GMP-compliant manufacturing [162]. An attractive alternative to based therapy is EVs that emerged as a promising cell-free therapeutic approach for immunomodulation. They also exhibit low immunogenicity and a favorable safety profile, while their small size and structure support stability, storage, and transport [161]. MSC-derived EVs may face significant barriers to clinical translation. Furthermore, EVs often have a short in vivo half-life and are rapidly cleared, limiting their therapeutic window. Storage at −80 °C alters their biological activity, and although freeze-drying is preferred for long-term preservation, it is logistically demanding and costly [163]. For example, systemically administered EVs exhibit a short half-life and are quickly cleared by mononuclear phagocytes, and their terminal half-life rarely exceeds 60 min [163]. Finally, depending on their cargo and the tumor microenvironment, MSC-EVs may exert pro-tumorigenic effects. This dual potential raises concerns about safety and predictability [164].

In summary, MSCs hold considerable potential to serve as an effective therapeutic tool, particularly in cancer treatment. Nonetheless, extensive research is still required to develop highly effective methods and therapies that are tailored to both the specific cancer type and the individual patient.

## 5. Clinical Trials of MSCs in Cancer Therapy

Therapeutic gene- or anti-cancer drug-loaded MSCs have shown remarkable anti-tumor effects in preclinical studies [61]. In recent years, numerous clinical trials have been registered to assess the safety and effectiveness of MSC-based therapies in cancer treatment. Their clinical approval is hampered due to the insufficient number of positive outcomes. However, there are early-phase clinical trials that have a chance of success and therefore could lead to a breakthrough in the field of cancer therapies using MSCs. It is important to recognize that, despite their numerous advantages, MSCs may also contribute to tumor progression and metastasis by promoting angiogenesis or suppressing anti-tumor immune responses [61]. The main challenges in clinical applications of MSCs include substantial heterogeneity arising from donor and tissue variability (BM-MSCs, AT-MSCs, UCMSCs). Their stability of stemness, differentiation potential, and expansion capacity also differ under varying culture conditions. Additional obstacles involve inconsistent homing efficiency, variable immune compatibility influenced by the inflammatory environment, and context-dependent secretion of bioactive factors that lead to unpredictable functional outcomes [159].

Search results using defined terms identified 69 studies registered on ClinicalTrials.gov up to December 2025. Currently, seven studies are actively recruiting, and three are not yet recruiting. The Table 4 shows clinical trials from recent years.

## 6. Limitations and Challenges

Although MSCs used as drug carriers show considerable potential, most studies remain at the preclinical stage. Compared to conventional chemotherapy, MSC-based cytostatic delivery offers advantages in drug distribution, selectivity, toxicity, and overall efficacy [77,166,167]. Nonetheless, several challenges and concerns persist. The role of MSCs in cancer progression remains controversial [168]. MSCs exhibit limited proliferative capacity, which restricts their therapeutic efficiency. Moreover, exosomes derived from MSCs, although promising, show limited physicochemical stability, complicating their storage and modification [64]. A further limitation is the relatively low drug-loading capacity of MSCs, which poses a serious technical barrier [169]. Therapeutic efficacy also depends on the homing and tropism capacity of MSCs, which is superior in freshly isolated cells compared with those expanded in culture. While advanced MSCs’ modifications offer prospects for clinical translation, several aspects require optimization to prevent potential complications [160]. Additional issues include the lifespan and viability of modified cells, tumorigenic risk associated with implantation, the source of donor cells, the target microenvironment, and immunocompatibility [170]. Genetic modification introduces further challenges, such as vector selection, which affects transfection efficiency. Viral vectors may elicit host immune responses or insertional mutagenesis leading to oncogenesis, whereas non-viral methods often cause higher cell mortality or transient transgene expression [156,157]. One of the most frequently discussed concerns is the potential of MSCs to promote tumor growth. MSCs can modulate immune cell activity by secreting factors such as TGF-β (transforming growth factor β), which inhibits T-cell proliferation, and IL-10, which suppresses pro-inflammatory cytokines. They may also secrete molecules that enhance tumor angiogenesis. This dual role of MSCs can be both therapeutically useful and a significant limitation. The most commonly described strategies to overcome obstacles associated with MSC-based cancer therapies include reducing MSC heterogeneity, enhancing MSC biological functions to achieve desired therapeutic effects, and cell-free strategies. The genetic modifications of MSC described in this article also represent promising systems for addressing MSC functionality. Engineering allows MSCs to be used in two ways. On the one hand, properly designed MSCs can destroy tumors by delivering cytokines, drugs, oncolytic viruses, therapeutic genes, and non-coding RNA. On the other hand, their natural immune-suppressing properties can also be harnessed to reduce inflammation induced by anticancer treatments [157].

Although innovative strategies may reduce the risks associated with the use of MSC, their dual nature remains an obstacle to clinical translation of MSC-based therapies [157,171].

## 7. Conclusions and Future Perspectives

MSCs possess properties that make them valuable tools in anticancer therapy. Their demonstrated features include immunomodulatory and immunosuppressive capacity, genetic stability, modulation of the tumor microenvironment, suitability as delivery vehicles, and potential for genetic modification with effective homing and tropism abilities. MSCs can serve not only as drug carriers but also in advanced strategies such as the delivery of oncolytic viruses and expression of anticancer proteins, miRNA, or therapeutic genes. Despite these advantages, several challenges remain. Future research must focus on minimizing damage to healthy tissues, improving MSCs’ survival, enhancing homing efficiency, and optimizing both the efficacy and standardization of MSC-based approaches [68]. Genetically modified MSCs show particularly high therapeutic potential and offer the possibility of personalized, targeted anticancer therapy. Preclinical in vivo and in vitro studies have demonstrated anticancer effects, including inhibition of tumor growth, suppression of proliferation and angiogenesis, induction of apoptosis, and enhancement of immune activation. Further investigations are essential to address remaining limitations and to enable the translation of MSC-based cancer therapies into clinical applications.

The application of artificial intelligence (AI) in the design of MSC-based therapies may become a cornerstone of future therapeutic strategies. Integrating AI with cell engineering opens new possibilities for therapy optimization by providing tools to standardize MSC modifications and increase their clinical efficacy. AI relies on machine learning (ML), which involves analyzing large datasets, recognizing patterns, and generating predictions and decisions. These technologies can substantially tailor personalized treatment plans based on individual patient profiles by integrating diverse datasets to precisely select treatments, improve efficacy, and reduce the risk of adverse events. As mentioned above, optimizing MSCs for specific therapeutic outcomes presents multiple challenges. AI, particularly through ML and deep learning (DL), is increasingly being used to address these challenges, offering powerful tools to increase the efficiency of MSC modification, including gene editing, functional optimization, quality control, predictive modeling, colony formation, and differentiation [172]. At the biological level, AI can be used to predict MSC differentiation, immunomodulatory function, and therapeutic potential by analyzing data, deciphering heterogeneity, and increasing precision. DL models based on MSC morphology can effectively predict differentiation propensity and uncover the regulatory networks underlying intrinsic heterogeneity. Furthermore, AI, through data-driven materials design (ML-based models), can correlate material parameters with biological properties, enabling the optimization of biomaterial research. However, the integration of these advanced technologies in clinical practice requires further validation to confirm their safety and effectiveness [173].

## Figures and Tables

**Table 1:** Studies on the use of MSCs as a carrier of specific drugs in various cancers.

Disease	Drug Used in the Study	Source of Used MSCs	Reduction of Cancer Cells Viability	Reference
Oral squamous cell carcinoma	Paclitaxel, Doxorubicin, Gemcitabine	Gingival papillae	>90%	[55]
Glioblastoma	Paclitaxel	Mouse bone marrow	~80%	[56]
Breast cancer	Doxorubicin	Adipose tissue	43.3%	[57]
Melanoma with lung metastases	Nanoparticles with doxorubicin	Adipose tissue (mouse model)	~55% (in vitro) ~60% (in vivo, metastases reduction)	[58]
Colon cancer	Doxorubicin	Mouse bone marrow	65%	[59]

**Table 2:** Comparison of key characteristics of MSCs derived from different tissue sources.

Feature	BM-MSCs [40,67,69,70]	AT-MSCs [18,67,69,71]	PB-MSCs [72,73,74,75]
Accessibility	Invasive and painful tissue harvesting procedures compared to UC-MSC	Widespread availability, facile acquisition with minimal tissue disruption	High accessibility, minimally invasive, accessible via blood banks, no ethical concerns
Isolation and expansion	Ease of isolating, remarkable ability for in vitro expansion and multi-lineage differentiation	Uncomplicated isolation protocols, high proliferative efficiency	Low percentage of MSCs in steady state—difficult isolation and expansion, PB-MSCs had lower proliferative capacity than BM-MSCs
Immunogenicity	Low immunogenicity results from the absence of HLA-DR expression	Low immunogenicity reducing the risk of immune rejection—low expression of MHC II	MHC II negative, which may suggest a low ability to induce an immune response
Homing efficiency	Inherent homing properties identified as key enabler for widespread use	A strong ability to specifically migrate towards the tumor microenvironment and inflammatory sites	PB-MSCs homing is faster than BM-MSCs
Drug-loading capacity	High uptake, effective absorption of doxorubicin, paclitaxel, gemcitabine, simple incubation	Similar capacity to BM-MSCs, confirmed absorption of anticancer drugs (cisplatin, paclitaxel)	No data

BM-MSCs—MSCs derived from bone marrow, AT-MSCs—MSCs derived from adipose tissue, PB-MSCs—MSCs derived from peripheral blood, HLA-DR—human leukocyte antigen—DR isotype, MHC II—major histocompatibility complex type II.

**Table 3:** Various types of miRNAs using in MSC-based cancer therapies and effects of their actions.

Type of miRNA	Effects of Actions	References
miR-34a	Fighting cancer stem cells, Relapse prevention; ↓ Tumor growth; ↓ Proliferation of cancer cells (50% after 72 h); ↑ DNA damage (6.5 times); ↓ Telomerase activity (~68%); ↑ Cancer cells’ senescence (percentage of senescent cells increased from ~8% to 72%) (glioma)	[130,131,132]
miR-let7	↓ Proliferation (10–30%) and migration of cancer cells (castration-resistant prostate cancer); ↓ Invasion (75–80%); ↓ Tumor growth (40%) (breast cancer, mouse model)	[133,134]
miR-124	↓ Invasion (75–80%) and migration (75%); ↓ EZH2 (oncogene) (65–75%) (pancreatic cancer); ↓ Immunosuppressive function of regulatory T cells, which enhances the immune response against cancer	[135,136]
miR-16	↓ Epithelial–mesenchymal transition; ↓ Invasion (50–70%); ↓ Tumor growth (tumor volume reduced by 50%, tumor weight by 47%) (breast cancer)	[137]
miR-342-3p	↓ Metastasis (~45%) and chemoresistance (breast cancer)	[138]
miR-551b-3p	↓ Cancer progression (breast cancer)	[138]
miR-21-5p	↓ Invasiveness (breast cancer)	[138]
miR-3182	↑ Apoptosis and ↓ invasiveness of (triple negative breast cancer)	[138]
miR-148b-3p	↓ Cancer progression (breast cancer)	[138]
miR-145	↑ Apoptosis (signal for cell death (tp53) was amplified 3–5 times) and ↓ metastasis (50-fold reduction in MMP9 levels) (breast cancer)	[139]

↑ induction, ↓ inhibition/reduction.

**Table 4:** Summary of clinical trials on the use of mesenchymal stromal/stem cells in cancer therapies.

Study ID	Phase	Study Title	Intervention	MSC Source/Type	Cancer Type	Objective	Status of Study	Reference
NCT04758533	1/2	AloCELYVIR in DIPG in combination with radio- or MB in monotherapy	BM-hMSCs + AdV 500.000 cells/kg, Weekly IVI/8 week	BM-hMSCs	Diffuse Intrinsic Pontine Glioma (DIPG), Medulloblastoma (MB)	Safety, Efficacy	Active, not recruiting	[69,165]
NCT03896568	1	MSC-DNX-2401 in treating patients with rHGG	MSCs + OAd DNX-2401, IA	BM-hMSCs	High-Grade Glioma	Best dose, Side effects, Toxicity, Capacity, DNX-2401 delivery to rHGG	Recruiting	[69,165]
NCT03298763	1/2	Targeted Stem Cells Expressing TRAIL as a Therapy for Lung Cancer	MSCTRAIL + pemetrexed/cisplatin	MSCs genetically modified to express TRAIL	Metastatic Non-small cell lung cancer (NSCLC)	Safety, Anti-tumor activity of MSCTRAIL in addition to CTX	Completed	[69,165]
NCT02530047	1	Mesenchymal Stem Cells (MSCs) for Ovarian Cancer	MSC-INFb, 10^5^ MSC/kg once a week for 4, IP	MSCs from healthy male donors and genetically changed	Ovarian cancer	Highest tolerable dose of MSC-INFb, Safety	Completed	[69,165]
NCT03608631	1	iExosomes in Metastatic Pancreas Cancer With KrasG12D Mutation	MSCs-DEs + KrasG12D siRNA, IVI over 15–20 min. on 1 d, 4 d, and 10 d, every 14 days for up to 3 cycles	Not defined	Metastatic pancreatic ductal adenocarcinoma (PDAC) with KrasG12D mutation	Best dose, Side effects	Recruiting	[69,165]
NCT05699811	1/2	IFNα Expressing MSCs for Locally Advanced/Metastatic Solid Tumors	MSC-IFNα + paclitaxel /cyclophosphamide/ anti-PD-1 monoclonal antibody, IVI 1–4 times every 4–6 weeks (2 × 10^6^ cells/kg) or 6 × 10^5^ cells/ kg, 2 × 10^5^/kg	hUC-MSCs	Locally advanced/metastatic ST: lung cancer, breast cancer, colorectal cancer, hepatocellular carcinoma, and sarcomas	Safety, Feasibility of MSC-IFNα	Recruiting	[165]
NCT04657315	1	Evaluation of MTD, safety, and efficiency of MSC11FCD therapy to rGBM (MSC11FCD-GBM)	MSCs + suicide gene, CD (MSC11 FCD), i.t. 1 × 10^7^, 3 × 10^7^ cells, Concomitant 5-FC 150 mg/kg/day	Not defined	Glioblastoma	MTD, Safety, Efficiency	Completed	[165]
NCT06446050	1	Chemokine and Costimulatory Molecule-modified MSCs for the treatment of aCRC	MSC-L, IV 1/2/3 × 10^6^ cells/kg, every 21 days for at least 6 cycles of treatment	hUC-MSCs	Colorectal cancer	Safety, Efficacy	Recruiting	[165]
NCT06890494	1	BiTE-EV therapy in Relapsed/ Refractory Acute B-Cell Lymphoblastic Leukemia	EVs with BiTEs, and CD3+/CD19+ MSCs. Take BiTE-EV every other day for 1 or 2 months	Culture supernatant with the bispecific vesicles BiTE-EV (CD3, CD19)	Acute B-cell lymphoblastic leukemia	Safety, Efficacy, MTD	Recruiting	[165]
NCT06245746	1	UCMSC-Exo in consolidation CTX-induced myelosuppression in AML	Single-time IVI	UCMSC-Exo	Acute Myeloid Leukemia	Safety, Efficacy	Recruiting	[165]
NCT05789394	1	Safety and preliminary efficacy of AMSCs for rGBM	IT received locally delivered AMSCs	AMSCs	Recurrent glioblastoma or astrocytoma	MTD, Safety, Preliminary efficacy	Recruiting	[165]
NCT07143812	1	Suicide gene expressing BM-hMSCs (MSC11FCD) in patients with newly diagnosed GBM	1 × 10^7^, 3 × 10^7^ cells IT or the tumor removal site using a syringe during surgery	BM-hMSCs	Glioblastoma	Safety, Tolerability, MTD	Not yet recruiting	[165]
NCT07048314	1/2	HB-adMSCs in the recovery of erectile function post radical retropubic prostatectomy of localized PCa	HB-adMSCs injected into the corpora cavernosa bilateral NVB, OR week 1 + 2.00 × 10^8^ via IV in clinic at week 12	HB-adMSCs	Prostate Cancer	Safety, Efficacy	Not yet recruiting	[165]
NCT06536712	1	IP EVs to prevent EAL in RC patients undergoing LAR	IP administered at the end of surgery	hPMSC-DE	Rectal Cancer	Safety, Efficacy	Not yet recruiting	[165]
NCT05672420	1/2	UC-MSCs in treatment-induced myelosuppression in HMs (USMYE Trial)	2 weeks of treatment: 5 dose-escalation levels and 3 frequency-escalation levels	UC-MSCs	Induced myelosuppression and acute myeloid leukemia/acute lymphoblastic leukemia	Safety, Efficacy	Completed	[165]

DIPG—Diffuse Intrinsic Pontine Glioma; MB—Medulloblastoma; CTX—Chemotherapy; IVI—Intravenous infusion, IA—Intra-arterial; IP—Intraperitoneal infusion; IT—Intratumoral; AdV—Adenovirus; BM-hMSCs—Allogeneic bone marrow-derived human mesenchymal stem cells; OAd—Oncolytic adenovirus; rHGG—Recurrent High-Grade Glioma; hMSCs-INFb—Human mesenchymal stem cells with interferon beta; MSCs-DEs—Mesenchymal stromal cells-derived exosomes; hUC-MSCs—Human umbilical-cord-derived mesenchymal stromal cells; ST—Solid tumors; MTD—Maximum Tolerated Dose; rGBM—Recurrent Glioblastoma; CD—Cytosine deaminase; 5-FC—5-Flucytosine; aCRC—Advanced Colorectal Cancer; MSC-L—Mesenchymal Stem Cells mobilizing Lymphocytes; EVs—Extracellular vesicles, UCMSC-Exo—Umbilical cord derived mesenchymal stem cells exosomes; AML—Acute myeloid leukemia; AMSCs—Allogenic adipose-derived mesenchymal stem cells; GBM—Glioblastoma; HB-adMSCs—Allogeneic adipose-derived mesenchymal stem cells; PCa—Prostate Cancer; NVB—Neuro-vascular bundle; OR—Operating room; hPMSC–DE—Human placenta mesenchymal stem cells derived exosomes; EAL—Early Anastomotic Leakage; RC—Rectal cancer; LAR—Low Anterior Resection; HMs—Hematological malignancies.

## Data Availability

No new data were created or analyzed in this study. Data sharing is not applicable to this article.

## Abbreviations

The following abbreviations are used in this manuscript:

aCRC	Advanced colorectal cancer
AdV	Adenovirus
AI	Artificial intelligence
Akt	Protein kinase B
AML	Acute myeloid leukemia
AMSC	Allogenic adipose-derived mesenchymal stem cell
AT-MSC	Adipose tissue cell
AVVs	Adeno-associated viruses
BM-MSC	Bone marrow cells
CAA	Cancer-associated adipocyte
CAF	Cancer-associated fibroblasts
circRNA	Circular RNA
CCL5	C–C motif chemokine ligand 5
CCR-2	C-C chemokine receptor type 2
dCas9	Catalytically deficient Cas9
CD	Cytosine deaminase
CRAd	Conditionally replicative adenovirus
CRISPR/ Cas	Clustered regularly interspaced short palindromic repeats–associated protein
CRT	Calreticulin
CSC	Cancer stem cell
CSF-1	Colony-stimulating factor 1
CTL	Cytotoxic T lymphocytes
CTXs	Chemotherapy
CXCL7	Chemokine (C-X-C motif) ligand 7
CXCL10	Chemokine (C-X-C motif) ligand 10
CXCR4	Chemokine receptor type 4
CXCR3	Chemokine receptor type 3
CXCR7	Chemokine receptor type 7
DAMPs	Damage-associated molecular patterns
DCs	Dendritic cells
DDS	Drug delivery system
DIPG	Diffuse intrinsic pontine glioma
DISC	Death-inducing signaling complex
DL	Deep learning
DOX	Doxycycline
DSBs	Double-stranded breaks
DR	Death receptor
EAL	Early anastomotic leakage
EGFR	Epidermal growth factor receptor
Epo	Erythropoietin
ERK	Extracellular signal-regulated kinase
ES	Endostatin
EVs	Extracellular vesicles
FADD	Fas-associated death domain
FGF	Fibroblast growth factor
GBM	Glioblastoma multiforme
GCV	Ganciclovir
GDEPT	Gene-directed enzyme prodrug therapy
gRNA	Guide ribonucleic acid
HB-adMSCs	Allogeneic adipose-derived mesenchymal stem cells
HIF-1α	Hypoxia-inducible factor 1
HLA-DR	Human leukocyte antigen—DR isotype
HMBG1	High-mobility group box 1
HMs	Hematological malignancies
hMSCs-INFb	Human mesenchymal stem cells with interferon beta
hPMSC–DE	Human placenta mesenchymal stem cell-derived exosome
HPV	Human papillomavirus
HSPs	Heat shock proteins
HSV	Herpes simplex virus
HSVTK	Herpes simplex virus thymidine kinase
hUC-MSC	Human umbilical-cord-derived mesenchymal stromal cell
IA	Intra-arterial
ICAM-1	Intracellular adhesion molecule-1
ICD	Immunogenic cell death
IFN	Interferon
IGF-1	Insulin-like growth factor 1
IL-6	Interleukin-6
IL-8	Interleukin-8
ILV	Intraluminal vesicles
LncRNA	Long non-coding RNA
IP	Intraperitoneal
IT	Intratumorally
IVI	Intravenous infusion
JAM-A	Junctional adhesion molecules-A
LAR	Low Anterior Resection
MAPK	Mitogen-activated protein kinase
MB	Medulloblastoma
MCP-1	Monocyte chemoattractant protein-1
MDC	Macrophage-derived chemokine
MHC II	Major histocompatibility complex type II
miRNA	Micro ribonucleic acid
ML	Machine learning
MMPs	Metalloproteinases
MMP-2	Matrix metalloproteinase-2
MMP-9	Matrix metalloproteinase-9
MSCs	Mesenchymal stromal/stem cells
MSCs-Des	Mesenchymal stromal cells-derived exosomes
MSC-L	Mesenchymal stem cell-mobilizing lymphocytes
MTD	Maximum tolerated dose
mTOR	Mammalian target of rapamycin
MV	Measles virus
MVB	Multivesicular bodies
Myo10	Myosin 10
MYXV	Myxoma virus
NDV	Newcastle disease virus
NK	Natural killer
NPs	Nanoparticles
NSCLC	Non-small cell lung cancer
NVB	Neuro-vascular bundle
OAd	Oncolytic adenovirus
OVs	Oncolytic viruses
OR	Operating room
PAMPs	Pathogen-associated molecular patterns
PB-MSCs	Peripheral blood cells
PCa	Prostate cancer
PDAC	Pancreatic ductal adenocarcinoma
PDGF	Platelet-derived growth factor
PDGF-AB	Platelet-derived growth factor-AB
PDK1	3-phosphoinositide-dependent protein kinase 1
PDL	Programmed death ligand
PGF	Placental growth factor
PI3K	Phosphoinositide-3-kinase
PSGL-1	P-selectin ligand
rGBM	Recurrent glioblastoma
rHGG	Recurrent high-grade glioma
RC	Rectal cancer
RISC	RNA-induced silencing complex
RNAi	RNA interfering
ROS	Reactive oxygen species
SDF-1	Stromal cell-derived factor-1
siRNA	Small interfering ribonucleic acid
ST	Solid tumors
STAT3	Signal transducer and activator of transcription 3
TAAs	Tumor-associated antigens
TA-MSCs	Tumor-associated mesenchymal stromal/stem cells
TGF-β	Transforming growth factor β
TIMPs	Tissue inhibitors of metalloproteinases
TME	Tumor microenvironment
TNF	Tumor necrosis factor
TNF-α	Tumor necrosis factor-alfa
TNTs	Tunneling nanotubes
TRAIL	Tumor necrosis factor-related apoptosis-inducing ligand
UCMSC-Exo	Umbilical cord-derived mesenchymal stem cells exosomes
VACV	Vaccinia virus
VEGFR1	Vascular endothelial growth factor receptor 1
VCAM-1	Vascular cell adhesion molecule-1
VLA-4	Very late antigen-4
WHO	World Health Organization
ZFNs	Zinc-finger nucleases
5-FC	5-flucytosine
